# Measuring Tuberculosis Medication Adherence: A Comparison of Multiple Approaches in Relation to Urine Isoniazid Metabolite Testing Within a Cohort Study in India

**DOI:** 10.1093/ofid/ofab532

**Published:** 2021-10-17

**Authors:** Ramnath Subbaraman, Beena E Thomas, J Vignesh Kumar, Maya Lubeck-Schricker, Amit Khandewale, William Thies, Misha Eliasziw, Kenneth H Mayer, Jessica E Haberer

**Affiliations:** 1 Department of Public Health and Community Medicine and Center for Global Public Health, Tufts University School of Medicine, Boston, Massachusetts, USA; 2 Division of Geographic Medicine and Infectious Diseases, Tufts Medical Center, Boston, Massachusetts, USA; 3 Department of Social and Behavioural Research, ICMR-National Institute for Research in Tuberculosis, Chennai, India; 4 Microsoft Research India, Bangalore, Karnataka, India; 5 The Fenway Institute, Fenway Health and Department of Medicine, Beth Israel Deaconess Medical Center and Harvard Medical School, Boston, Massachusetts, USA; 6 Center for Global Health, Massachusetts General Hospital and Department of Medicine, Harvard Medical School, Boston, Massachusetts, USA

**Keywords:** tuberculosis, medication adherence, measurement, India, drug metabolite testing

## Abstract

**Background:**

Nonadherence to tuberculosis medications is associated with poor outcomes. However, measuring adherence in practice is challenging. In this study, we evaluated the accuracy of multiple tuberculosis adherence measures.

**Methods:**

We enrolled adult Indians with drug-susceptible tuberculosis who were monitored using 99DOTS, a cellphone-based technology. During an unannounced home visit with each participant, we assessed adherence using a pill estimate, 4-day dose recall, a last missed dose question, and urine isoniazid metabolite testing. We estimated the area under the receiver operating characteristic curve (AUC) for each alternate measure in comparison to urine testing. 99DOTS data were analyzed using patient-reported doses alone and patient- and provider-reported doses, the latter reflecting how 99DOTS is implemented in practice. We assessed each measure’s operating characteristics, with particular interest in specificity—that is, the percentage of participants detected as being nonadherent by each alternate measure, among those who were nonadherent by urine testing.

**Results:**

Compared with urine testing, alternate measures had the following characteristics: 99DOTS patient-reported doses alone (area under the curve [AUC], 0.65; specificity, 70%; 95% CI, 58%–81%), 99DOTS patient- and provider-reported doses (AUC, 0.61; specificity, 33%; 95% CI, 22%–45%), pill estimate (AUC, 0.55; specificity, 21%; 95% CI, 12%–32%), 4-day recall (AUC, 0.60; specificity, 23%; 95% CI, 14%–34%), and last missed dose question (AUC, 0.65; specificity, 52%; 95% CI, 40%–63%).

**Conclusions:**

Alternate measures missed detecting at least 30% of people who were nonadherent by urine testing. The last missed dose question performed similarly to 99DOTS using patient-reported doses alone. Tuberculosis programs should evaluate the feasibility of integrating more accurate, objective measures, such as urine testing, into routine care.

Medication adherence is a critical aspect of tuberculosis (TB) care [1–3]. In clinical trials, missing >10% of doses during TB therapy is associated with ~6 times increased risk of poor outcomes [2]. Similarly, in India’s TB program, poor adherence has been associated with higher unfavorable treatment outcomes and disease recurrence [3, 4].

Many TB programs have historically monitored adherence using directly observed therapy (DOT), often using a facility-based approach, in which people with TB (PWT) visit clinics where health care providers (HCPs) watch them take medications. In light of growing concerns about the ethics, feasibility, and effectiveness of DOT [5–9], TB programs in India, China, and other countries have increasingly shifted toward use of digital adherence technologies (DATs) or self-administered therapy (SAT)—that is, PWT taking medications themselves at home or in another preferred setting [10, 11]. Shifts by TB programs away from DOT have only accelerated in the context of the coronavirus disease 2019 (COVID-19) pandemic, as in-person observation of dose ingestion has become more challenging [12].

With the transition away from DOT, HCPs face challenges in measuring adherence. In the context of SAT, self-reported measures and pill counts frequently overestimate adherence due to social desirability bias [13, 14]. While DATs are felt to be more objective, research in TB [15–18] and other diseases [19] has revealed inaccuracies in measurement by DATs that vary by technology and population. Even the accuracy of DOT may be limited by noningestion of doses, difficulty observing weekend doses, and incomplete observation by HCPs. Understanding the benefits and limitations of various adherence measures may help TB programs integrate the most useful approaches into clinical care to identify and address nonadherence.

In comparison to other diseases [20, 21], little research has evaluated the accuracy of adherence measures in TB care, perhaps due to the dominance of DOT. One high-quality prior study conducted in Tanzania used the Medication Event Monitoring System (MEMS; a digital pillbox) to evaluate operating characteristics of other measures [22]. This study found that concurrent use of multiple clinic-based adherence measures facilitated identification of most PWT who were nonadherent by MEMS. However, these approaches misclassified many PWT with high adherence, which was common in the cohort (>96% of doses were estimated to have been taken). The study was further limited by a small sample (50 participants) and clinic-based assessments, such that participants may have altered their behavior in anticipation of study interactions.

In this manuscript, we compared multiple measures of adherence to drug-susceptible TB therapy through secondary analysis of a cohort study conducted in India. Primary findings from this cohort study have been published previously [4, 15]. We conducted a single unannounced home visit for each participant, during which we collected a urine sample that was tested for isoniazid metabolites. By comparison to this objective indicator of medication ingestion, we assessed the operating characteristics of 4 alternate adherence measures: 99DOTS (a cell phone–based DAT), a pill estimate, 4-day dose recall, and a question assessing timing of the last missed dose.

## METHODS

### Participant Consent

Written consent was obtained from participants. This study was approved by ethics committees at Tufts University (Boston, MA, USA), Brigham and Women’s Hospital (Boston, MA, USA), and the Indian Council of Medical Research (ICMR)–National Institute for Research in TB (NIRT; Chennai, India).

### Study Setting

We recruited PWT from 3 cities with a high TB burden [23, 24]. In Chennai and Vellore, we recruited people with HIV (PWH) who were undergoing TB treatment at these cities’ 5 largest HIV antiretroviral therapy (ART) centers. In Mumbai, we recruited HIV-negative PWT from 11 DOT centers selected for their high patient volumes.

### Participant Recruitment and Data Collection

During August 2017 to February 2019, we sequentially recruited people with drug-susceptible TB who were ≥18 years of age and eligible for 99DOTS monitoring [4, 15]. Participants were recruited during clinic visits to start TB treatment or collect medication refills, excluding those in the last treatment month (to allow time for study procedures). Using this approach, we exhausted the pool of individuals who were already taking treatment. We continued to enroll participants who were starting treatment but randomly chose their home visit to occur in the first 2 treatment months (intensive phase) or the last 4 months (continuation phase) to ensure representation of home visits across phases.

At enrollment, we consented participants for a future unannounced home visit and administered a socio-behavioral questionnaire [4]. We conducted the home visit at least 3 weeks after enrollment, or after the start of the continuation phase for participants randomized to undergo the home visit in that phase. The exact visit day was selected using a random number generator.

The home visit was conducted without prior notice to minimize changes in adherence behavior in anticipation of study interactions (“Hawthorne effect” [25]). At the visit, we administered an adherence questionnaire and collected a urine sample for isoniazid metabolite testing. Although the single visit limits understanding of adherence throughout treatment for individual participants, the visits provide information distributed throughout the treatment course for the sample as a whole [4].

### Interpretation of Urine Isoniazid Test Results

We used IsoScreen, a validated urine test that objectively measures recent TB medication ingestion, as the comparator against which alternate adherence measures were evaluated (hereafter, “the urine test”) [26–28]. If the participant’s urine contains isoniazid metabolites, the test reagents turn purple/blue or green. Purple/blue suggests that a dose was taken in the last 24 hours. Green suggests that a dose was taken 24–48 hours previously [26, 27]. A yellow result (no color change) suggests that a dose has not been taken for at least 48–72 hours (supplementary text, Supplementary Table 1). IsoScreen has been shown to have relatively high but imperfect inter-rater agreement in interpretation of results [29]; however, we minimized risk of variable interpretation by having field researchers bring urine samples back to the NIRT lab (in Chennai) or a designated clinic (in Mumbai and Vellore), where the test was run and the color result agreed upon by both a lab technician and the field researcher. As India’s national TB program uses fixed-dose combination pills, isoniazid adherence also serves as a proxy for other medications in the drug-susceptible TB regimen.

Based on these color changes, we defined poor adherence by urine testing using 2 approaches (Table 1). We defined “nonadherence” as comprising a yellow result (compared with a purple/blue or green result), which suggests missed doses for 72 hours or more. We defined “suboptimal adherence” as comprising a yellow or green result (compared with a purple/blue result), which suggests missed doses for 48 hours or more. While prior analyses from this cohort showed that both nonadherence and suboptimal adherence were associated with unfavorable TB treatment outcomes [4], our primary analyses focus on nonadherence, because suboptimal adherence is slightly more likely to misclassify adherence (ie, having taken a dose within the last 24 hours). We present parallel analyses for suboptimal adherence in the supplementary text and tables.

**Table 1.** Approaches to Measuring Tuberculosis Medication Adherence and Interpretation of Data From These Measures

Adherence Measure	Manufacturer or Source of the Measure	Description of the Measurement Approach	Classification of Data From Adherence Measures as Nominal, Ordinal, or Continuous Variables	Classification of Data From Adherence Measures as Binary Variables
IsoScreen (urine test for isoniazid metabolites)	GFC Diagnostics, United Kingdom (based on the Arkansas method for urine isoniazid testing) [26, 27, 30]	The PWT’s urine is injected using a syringe into a vial containing reagents that change color if isoniazid metabolites are present. Color changes indicate the time of last medication ingestion: purple/blue (<24 hours ago), green (24–48 hours ago), and yellow (>48 hours ago).	N/A	We used 2 binary interpretations: (1) purple/blue or green (adherence) vs yellow; (nonadherence) (2) purple/blue (adherence) vs green or yellow (suboptimal adherence).
99DOTS	Everwell Health Solutions, India [10]	Medication blister packs are dispensed in a custom envelope. Dispensing a daily dose reveals a hidden number on the envelope that the PWT calls for free. If a phone call is made on a given day, the dose is logged as having been taken in the electronic dosing history (“patient-reported dose”). If a PWT misses reporting doses, a health care provider contacts the PWT and then reports doses themselves based on discussion with the PWT (“provider-reported doses”).	Using the call record for the 2 days preceding and up to 6 hours before the home visit on the day of the visit, an ordinal variable was created ranging from 0 to 3, representing the number of days the PWT and/or provider did not call to report a dose taken (ie, “nonengagement”).	0–1 day of nonengagement vs 2–3 days of nonengagement (this same breakdown was used for patient-reported doses alone and patient- and provider-reported doses).
Pill estimate	—	Relative to the PWT’s last medication refill date, field researchers qualitatively assessed whether doses appeared taken as expected, or whether there was an unexpected shortage or excess of pills.	A 3-category nominal variable was used corresponding to whether pills were taken as expected, in excess, or in shortage.	Pills taken as expected vs shortage or excess of pills
4-day recall	Adapted from the ACTG adherence follow-up questionnaire [31]	PWT are asked to recall whether they took their medication doses in the 4 previous days, including the approximate time of dose ingestion.	Excluding the day of the home visit, an ordinal variable was created ranging from 0 to 4, representing the number of reported missed doses in the previous 4 days.	0 doses missed vs 1 to 4 doses missed in the previous 4 days
Last missed dose question	Adapted from the ACTG adherence baseline questionnaire [31]	PWT are asked when they last missed taking any medication doses, with the following item responses: never skipped, within the past week, 1–2 weeks ago, 2–4 weeks ago, 1–3 months ago, and >3 months ago.	A 6-category nominal variable was created corresponding to each of the item responses.	Never skipped a dose vs dose missed any time during treatment

Abbreviations: ACTG,AIDS Clinical Trials Group; PWT, people with tuberculosis.

Based on prior studies that compared the urine test to directly observed dosing, the sensitivity of our definition of nonadherence—that is, the percentage of participants classified as being adherent by urine testing among those who truly took a dose within the last 24 hours—is >99%. The specificity of our definition of nonadherence—that is, the percentage of participants classified as being nonadherent by urine testing among those who have truly not taken doses for 72 hours—is 88%, suggesting that there is a <12% chance of misclassifying participants who did not take any dose within the prior 72 hours as being adherent [26, 27].

### Interpretation of Alternate Adherence Measures

We also collected data using 4 alternate adherence measures. As described in Table 1, 99DOTS captured data longitudinally, while the other measures were assessed during the home visit. 99DOTS has been used to monitor >200 000 PWT in India’s TB program [10]. 99DOTS’ electronic record captures doses reported by daily phone calls from PWT (ie, “patient-reported doses”). If a PWT does not call for a day or more, HCPs are supposed to contact the PWT and report whether these doses were taken based on the PWT’s verbal report (ie, “provider-reported doses”). We analyzed 99DOTS’ operating characteristics separately for patient-reported doses alone and for both patient- and provider-reported doses [4].

For the pill estimate, researchers observed each participant’s medication blister packs and qualitatively reported whether pills were “taken as expected” or whether there was a “shortage” or “excess” in relation to the last refill date. Pill shortage could represent nonadherence from delayed refill collection, while pill excess could suggest skipped daily doses. While we did not quantify the exact number of remaining pills, our goal was to use a rapid approach that HCPs could replicate in routine care. Questions assessing when the participant last missed a dose (“last missed dose question”) and recall of doses taken in the previous 4 days (“4-day recall”) were adapted from a standardized HIV adherence questionnaire [31].

### Analyses

Analyses were conducted using Stata SE 16.1. We analyzed operating characteristics of the alternate adherence measures using 2 approaches: the predictive approach and the dose date and time correspondence (DDTC) approach. The predictive approach utilized the full range of responses for each adherence measure and combined measures to evaluate how they predicted nonadherence by urine testing. Adherence measures captured data as ordinal (99DOTS, 4-day recall) or nominal (pill estimate, last missed dose question) variables (Table 1). Although 99DOTS captured data from the time when participants started using the technology, we used call records for the 2 days preceding the home visit and the day of the visit (inclusive of up to 6 hours before the visit time). This approach provided a standard denominator of data that was concurrent with the time period measured by the urine test. We also explored the use of 14 days of call data before the home visit, but it did not improve 99DOTS’ operating characteristics above using 3 days of call data. A small number of enrolled participants were initiated on 99DOTS after the home visit date and were excluded from the analysis of the technology’s accuracy.

To estimate sensitivity, specificity, positive predictive value (PPV), and negative predictive value (NPV), we transformed ordinal or nominal variables for each adherence measure into binary variables as follows. First, we assessed prevalence ratios of nonadherence by urine testing for each variable category in relation to the lowest risk category. For example, for 4-day recall, we assumed that reporting missing 0 doses would be associated with the lowest risk. Using this reference category, we then assessed prevalence ratios of nonadherence by urine testing if participants reported missing 1, 2, 3, or 4 doses.

To create binary variables, we primarily assessed how each adherence measure might practically be used in routine care to identify potential nonadherence. For example, for the last missed dose question, we classified “no missed doses” as indicating likely adherence, while report of missing doses anytime during treatment was classified as potential nonadherence. We secondarily grouped categories that had a statistically significant increase in prevalence ratios of nonadherence by urine testing, in comparison with categories that did not have an increase in prevalence ratios.

We evaluated the area under the receiver operating characteristic curve (AUC) for each measure when analyzed as a nominal, ordinal, or binary variable in relation to nonadherence by urine testing. We used binary variables to estimate sensitivity, specificity, PPV, and NPV in relation to nonadherence by urine testing (Table 2).

**Table 2.** Definitions for the Operating Characteristics of the Adherence Measures in This Study. Definitions Are Provided for the Outcome of Nonadherence According to the Urine Test, With Relevant Modifications for the Outcome of Suboptimal Adherence Clarified in the Footnotes

Operating Characteristic	Definition
Sensitivity	Percentage of participants who were classified as being adherent by an alternate measure among those who were adherent by the urine test (ie, purple/blue or green result)^a^
Specificity	Percentage of participants who were classified as being nonadherent by an alternate measure among those who were nonadherent by the urine test (ie, yellow result)^b^
Positive predictive value	Likelihood of a participant being urine test–adherent^a^ among those classified as being adherent by an alternate measure
Negative predictive value	Likelihood of a participant being urine test–nonadherent^b^ among those classified as being nonadherent by an alternate measure

For analyses using the outcome of suboptimal adherence, adherence was defined as a purple/blue result only.

For analyses using the outcome of suboptimal adherence (see the supplementary text and tables), suboptimal adherence was defined as a yellow or green result.

The DDTC approach, which we previously used to evaluate 99DOTS [15], aimed to precisely assess whether the reported adherence history in the days before the home visit reflected adherence by urine testing. Because only 99DOTS and 4-day recall captured dosing dates and times, we describe the DDTC analysis findings in the supplementary text and tables.

## RESULTS

### Participant Characteristics

Of participants screened at our study sites, 832 met eligibility criteria, 84 (10%) of whom were not enrolled because a family member collected their medications (31 participants) or they did not consent to participate (53 participants). Despite 3 attempts, we were not able to complete home visits for 98 (13%) of the 748 enrolled participants. In the final analysis, 650 participants were included, of whom 77 (11.8%) were nonadherent and 116 (17.8%) were suboptimally adherent by urine testing. The median age (interquartile range) was 35 (25–45) years, 271 (42%) were female, and 303 (47%) were PWH. Other cohort characteristics have been described previously [4].

### Prevalence Ratios of Alternate Adherence Measures in Relation to Nonadherence by Urine Testing

Participants with 3 days of nonengagement (for 99DOTS patient-reported doses alone), 2 and 3 days of nonengagement (for 99DOTS patient- and provider-reported doses), and observed pill shortage (for the pill estimate) had statistically significantly increased prevalence ratios of nonadherence by urine testing (Table 3). In general, the magnitude of the association with nonadherence by urine testing was greater with higher reported missed doses (for 4-day recall) and the more recently participants reported missing doses (for the last missed dose question). Findings were similar for the outcome of suboptimal adherence by urine testing (Supplementary Table 2).

**Table 3.** Prevalence Ratios of Alternate Adherence Measures in Relation to the Outcome of Nonadherence by Urine Testing^a^ and Area Under the Receiver Operating Characteristic Curve for Each Measure

Alternate Adherence Measure	Nonadherent Participants by the Urine Test,^b^ No. (%)	Prevalence Ratio^c^ (95% CI)	*P* Value	Area Under the Receiver Operating Characteristic Curve^d^
99DOTS patient-reported doses alone (n=608)^e^				0.66
0 days nonengagement	9/138 (6.5)	Ref		
1 day nonengagement	11/211 (5.2)	0.8 (0.3–1.9)	.607	
2 days nonengagement	10/68 (14.7)	2.3 (1.0–5.3)	.062	
3 days nonengagement	37/191 (19.4)	3.0 (1.5–6.0)	.002∗	
99DOTS patient- and provider-reported (n=608)^e^				0.62
0 days nonengagement	28/314 (8.9)	Ref		
1 day nonengagement	17/212 (8.0)	0.9 (0.5–1.6)	.718	
2 days nonengagement	5/14 (35.7)	4.0 (1.8–8.8)	.001∗	
3 days nonengagement	17/68 (25.0)	2.9 (1.6–4.8)	<.001∗	
Pill estimate (n=650)				0.55
Taken as expected	61/570 (10.7)	Ref		
Excess of pills	12/67 (17.9)	1.7 (1.0–2.9)	.074	
Shortage of pills	4/13 (30.8)	2.9 (1.2–6.7)	.015∗	
4-day recall (n=650)				0.60
0 doses missed	59/614 (9.6)	Ref		
1 dose missed	8/19 (42.1)	4.4 (2.5–7.8)	<.001∗	
2 doses missed	4/9 (44.4)	4.6 (2.1–10.0)	<.001∗	
3 or 4 doses missed	6/8 (75.0)	7.8 (4.9–12.5)	<.001∗	
Last missed dose question (n=650)				0.67
Never skip medications	37/483 (7.7)	Ref		
Within the past week	19/47 (40.4)	5.3 (3.3–8.4)	<.001∗	
1–2 weeks ago	5/34 (14.7)	1.9 (0.8–4.6)	.14	
2–4 weeks ago	8/40 (20.0)	2.6 (1.3–5.2)	.007∗	
1–3 months ago	7/35 (20.0)	2.6 (1.3–5.4)	.010∗	
>3 months	1/11 (9.1)	1.2 (0.2–7.9)	.859	

Abbreviations: AUC, area under the curve; Ref,reference group.

Indicates statistical significance at the 5% level.

Nonadherence by urine testing was defined as a yellow urine test result, as compared with a green or purple/blue result, which comprised adherence.

Number of nonadherent patients by urine testing divided by the number of patients in each alternate measure category.

Prevalence ratio refers to a ratio of proportions—that is, the proportion of participants with nonadherence by urine testing in each category over the proportion of participants with nonadherence by urine testing in the reference category.

AUCs are in relation to the categorical breakdown of variables presented in the table.

We excluded 42 participants who were eligible for 99DOTS and recruited into the study, but whose 99DOTS enrollment date in the electronic system was after the home visit date.

### Accuracy and Operating Characteristics of the Alternate Adherence Measures

For nonadherence by urine testing, 99DOTS using patient-reported doses alone and the last missed dose question had the highest AUCs, regardless of whether they were considered as ordinal or nominal variables (Table 3) or binary variables (Table 4). For nonadherence by urine testing, 99DOTS using patient-reported doses alone had the highest specificity, followed by the last missed dose question, while the pill estimate and 4-day recall had considerably lower specificity (Table 4). In contrast, 4-day recall had the highest sensitivity, followed by the pill estimate and last missed dose question, while 99DOTS using patient-reported doses alone had the lowest sensitivity. Adding 4-day recall and the pill estimate to the last missed dose question mildly increased specificity while decreasing sensitivity. Findings regarding the AUCs, sensitivity, and specificity of these measures were similar for suboptimal adherence by urine testing (Supplementary Tables 2 and 3). Findings of the DDTC analyses are presented in the supplementary text and Supplementary Table 4.

**Table 4.** Operating Characteristics of Alternate Tuberculosis Medication Adherence Measures^a^ as Compared With Nonadherence by Urine Testing

Sample	No. of Participants in Sample	Sensitivity (95% CI), %	Specificity (95% CI), %	Positive Predictive Value (95% CI), %	Negative Predictive Value (95% CI), %	Area Under the Receiver Operating Characteristic Curve
99DOTS patient-reported doses alone	608	61 (57–65)	70 (58–81)	94 (91–96)	18 (14–23)	0.65
99DOTS patient- and provider-reported doses	608	89 (86–91)	33 (22–45)	91 (89–94)	27 (18–38)	0.61
Pill estimate	650	89 (86–91)	21 (12–32)	89 (86–92)	20 (12–30)	0.55
4-day recall	650	97 (95–98)	23 (14–34)	90 (88–93)	50 (33–67)	0.60
Last missed dose question	650	78 (74–81)	52 (40–63)	92 (90–95)	24 (18–31)	0.65
Last missed dose question and 4-day recall	650	77 (73–81)	55 (43–66)	93 (90–95)	24 (18–31)	0.65
Last missed dose question and pill estimate	650	73 (69–76)	56 (44–67)	92 (90–95)	22 (16–28)	0.64
Last missed dose question and 4-day recall and pill estimate	650	72 (68–76)	58 (47–70)	93 (90–95)	22 (16–28)	0.65

For this analysis, alternate adherence measures were classified as binary variables, as described in Table 1.

## DISCUSSION

We report the findings of the largest study to date comparing operating characteristics of different approaches for measuring TB medication adherence in comparison with urine testing, which is a rigorous, objective marker of recent isoniazid ingestion. Our study provides insights into benefits and limitations of different measures, including 99DOTS, a DAT that has been rolled out to monitor >200 000 PWT in India, Uganda, and other settings [10, 32, 33]. Notably, a single question evaluating the timing of participants’ last missed dose had comparable accuracy (ie, AUC) with 99DOTS using patient-reported doses alone. The last missed dose question had a higher AUC and higher specificity than 99DOTS using patient- and provider-reported doses, which represents how the technology is typically used in routine care [10]. Our findings also reveal how self-reported adherence, including via DATs, may be limited by socially desirable responses. Among participants with nonadherence by urine testing, <70% were classified as being nonadherent by every alternate measure, suggesting that better measures are needed.

99DOTS using patient-reported doses alone had the highest specificity for detecting participants with nonadherence by urine testing; however, other operating characteristics had limitations that may reduce 99DOTS’ benefits for adherence monitoring. For example, when using patient-reported doses alone, 99DOTS had the lowest sensitivity for appropriately classifying participants who were adherent by urine testing and the lowest NPV of all measures. As shown in a previous qualitative study, the low NPV likely results from PWT taking medications correctly but being unable or unwilling to call 99DOTS due to suboptimal cellular signal, phone access, or technology fatigue [16]. This NPV suggests that, for every 10 PWT whom HCPs contact because they are not engaging optimally with 99DOTS, only 2 PWT might actually be nonadherent. In addition, inclusion of provider-reported doses lowered 99DOTS’ specificity and AUC, which is concerning as this analysis represented how 99DOTS operates in routine care, as noted above. This finding suggests that, even after contacting PWT with low 99DOTS engagement, HCPs may still miss detecting a considerable proportion of PWT with nonadherence because these PWT provide socially desirable responses [15].

The operating characteristics of the single question regarding the last missed dose are notable. In contrast with 4-day recall, the last missed dose question provided a broader time window over which participants could admit to missing doses. Not surprisingly, participants who reported missing doses within the preceding week had a higher prevalence ratio for nonadherence by urine testing; however, so did participants who reported last missing doses 2–4 weeks or 1–3 months ago. In other words, reporting missed doses in the past was associated with current nonadherence. These participants may have felt it was more socially acceptable to report missing doses in the past, which could explain why this question identified considerably more nonadherent participants than 4-day recall. Although the last missed dose question had a low NPV, participant reports of last missing doses in the past (eg, weeks or months ago) may still provide important information. As shown in the HIV literature [34], social desirability is often less relevant when participants report nonadherence; that is, reported missed doses were likely truly missed. HCPs can use this information to provide enhanced support.

In contrast with the last missed dose question, 4-day recall had poor specificity, detecting only about one-fifth of nonadherent participants according to the urine test. The difference in specificity between these 2 self-report measures provides insights into how the structure of questions and the underlying cognitive psychology could increase or decrease socially desirable responses [35]. When asked about their recent dose-taking behavior with 4-day recall, most participants who were nonadherent by urine testing—8 out of 10—provided socially desirable responses indicating that they had been correctly taking their pills.

The pill estimate also had low specificity and AUC; however, these limitations may be related to our qualitative assessment of remaining pills, which anticipated approaches HCPs might use during busy clinic visits. Future studies using quantitative pill counts may clarify the accuracy that could be achieved with this measure; however, detailed pill counts may be difficult to implement in routine care and may be limited by PWT discarding pills to hide nonadherence [36]. Notably, a pill shortage was associated with higher nonadherence by urine testing. Based on insights from a previous study, this finding may reflect challenges PWT face in collecting medication refills, owing to the time, money, and transportation required to reach clinics [4]. As such, PWT commonly run out of pills, highlighting how structural barriers adversely impact medication adherence.

The most important finding of this study is that no alternate measure appropriately classified >70% of participants who were nonadherent by urine testing. As such, better adherence measures are needed. Integrating urine isoniazid testing into routine care might improve identification of nonadherence and facilitate interventions to improve subsequent TB outcomes, especially given that urine test results have been shown to be associated with TB treatment outcomes [4]. For this study, IsoScreen kits were procured internationally at a cost of ~USD$10 per test; however, given that this test is based on the decades-old Arkansas method, which involves simple chemical reagents, it may be possible to routinely conduct this test in laboratories in high–TB burden countries at a much lower cost [28]. A 1997 study conducted in the United Kingdom estimated that test reagents only cost about USD$0.06, which is equivalent to ~USD$0.10 (Indian rupees 7) today, adjusted for inflation [26].

Further research is needed to understand measurement of TB medication adherence. We need to better understand the urine test’s acceptability, feasibility, and cost-effectiveness in routine practice. Notably, 1 study found that urine tenofovir testing was highly acceptable to individuals for monitoring adherence to HIV preexposure prophylaxis [37]. Counseling strategies should be developed to minimize the risk that HCPs will stigmatize PWT with negative urine test results, as judgmental feedback can result in PWT disengaging from care [38]. Additionally, as our study assessed adherence at unannounced home visits, future studies should evaluate whether urine tests conducted at clinic visits provide comparable adherence information and predict treatment outcomes [39]. Future studies should also evaluate other adherence measures, including delayed medication refill visits, which may suggest that PWT have missed doses or are at risk for loss to follow-up. Acting upon such delays may be of benefit in India, where PWT face challenges in reaching clinics to collect their medication refills [4]. Measuring drug metabolites in other specimens such as hair may also be valuable, although these assays may be more costly than urine testing [40]. In addition, given that ~11% of people with TB in India without a prior treatment history (ie, “new cases”) have isoniazid-monoresistant disease [41], point-of-care drug metabolite testing approaches need to be developed for other TB drugs.

One limitation of our study is that operating characteristics vary depending on the cutoff used to dichotomize each adherence measure; however, AUCs were similar regardless of whether each adherence measure was evaluated as a multicategory variable (Table 3) or a binary variable (Table 4). Another limitation is that urine testing may not detect <12% of PWT who have not taken any doses for 72 hours; however, this limitation does not undermine our main finding, which is that alternate measures are less specific than the urine test for detecting nonadherence.

## CONCLUSIONS

In this cohort study of people with drug-susceptible TB, we found that the last missed dose question and 99DOTS using patient-reported doses alone had the highest accuracy (ie, AUC) of the adherence measures evaluated. Although 99DOTS using patient-reported doses alone detected the highest proportion of participants with nonadherence by urine testing, due to the technology’s very low NPV and low specificity when provider-reported doses were included, HCPs may face substantial practical challenges in identifying PWT with poor adherence. By comparison, the last missed dose question had similar accuracy, detected half of participants with nonadherence by urine testing, and involves negligible PWT and HCP burden. While the last missed dose question also had low NPV, reports of missed doses in the past may also indicate challenges faced by PWT that merit enhanced counseling. Finally, none of the alternate measures detected >70% of PWT who were nonadherent by urine testing. Further research is needed to integrate more accurate, objective adherence measures into routine TB care, potentially including urine testing or measurement of drug metabolites in other specimens.

## Supplementary Data

Supplementary materials are available at *Open Forum Infectious Diseases* online. Consisting of data provided by the authors to benefit the reader, the posted materials are not copyedited and are the sole responsibility of the authors, so questions or comments should be addressed to the corresponding author.

ofab532_suppl_Supplementary_Tables_S1-S4

ofab532_suppl_Supplementary_Materials_S1
