# Supplementary material for: Measuring Tuberculosis Medication Adherence: A Comparison of Multiple Approaches in Relation to Urine Isoniazid Metabolite Testing Within a Cohort Study in India
Source: Open Forum Infect Dis. 2021 Oct 17;8(11):ofab532. doi: 10.1093/ofid/ofab532 (PMC9088502; doi:10.1093/ofid/ofab532)
Supplement: ofab532_suppl_Supplementary_Tables_S1-S4 [file ofab532_suppl_Supplementary_Tables_S1-S4.docx]

**Supplementary Tables**

**Supplement to:**

*Measuring tuberculosis medication adherence: a comparison of multiple approaches in relation to urine isoniazid metabolite testing within a cohort study in India*

**Authors:** Ramnath Subbaraman, Beena E. Thomas, J. Vignesh Kumar, Maya Lubeck-Schricker, Amit Khandewale, William Thies, Misha Eliasziw, Kenneth H. Mayer, Jessica E. Haberer

**Correspondence:** Beena E. Thomas, Department of Social and Behavioural Research, ICMR- National Institute for Research in Tuberculosis, No. 1, Mayor Sathiyamoorthy Road, Chetpet, Chennai – 600 031, India ([beenaelli09@gmail.com](mailto:beenaelli09@gmail.com)). Phone: +91-44-2836-9525

**Table S1. Findings from two studies that evaluated operating characteristics of urine testing based on color changes in the assay at different time intervals after last ingestion of isoniazid**

|  | Soobratty et al. 2014 [9] | | | Elizaga et al. 1997 [8] | | | Both studies combined | | |
| --- | --- | --- | --- | --- | --- | --- | --- | --- | --- |
| Time after last dose | Purple/ blue color  N (%) | Green color  N (%) | Yellow color  N (%) | Purple/ blue color  N (%) | Green color  N (%) | Yellow color  N (%) | Purple/ blue color  N (%) | Green color  N (%) | Yellow color  N (%) |
| 24 hours | 86/105 (82) | 18/105 (17) | 1/105 (1) | 25/25 (100) | 0/25 (0) | 0/25 (0) | 111/130 (85) | 18/130 (14) | 1/130 (0.8) |
| 48 hours | 5/105 (5) | 84/105 (80) | 16/105 (15) | 19/25 (76)^a^ | | 6/25 (24) | 108/130 (83)^a^ | | 22/130 (17) |
| 72 hours | 0/104 (0) | 14/104 (13) | 90/104 (87) | 0/25 (0) | 1/25 (4) | 24/25 (96) | 0/129 (0) | 15/129 (12) | 114/129 (88) |

^a^Elizaga et al. 1997 did not differentiate between purple/blue and green color results for the 48 hour urine test results; however, based on the 36 hour findings reported in the paper, it seems likely that the vast majority of the results at 48 hours were green color.

**Table S2. Prevalence ratios of alternate adherence measures in relation to the outcome of suboptimal adherence by urine testing^a^ and each measure’s area under the receiver operating characteristic curve**

| **Adherence measure** | **Suboptimally adherent patients^b^**  **n (%)** | **Prevalence ratio**^c^ **(95% Confidence Interval)** | ***P* value** | **Area under the receiver operating characteristic curve (AUC)**^d^ |
| --- | --- | --- | --- | --- |
| **99DOTS patient-reported doses alone (N=608)**^e^ |  |  |  | 0.68 |
| 0 days nonengagement | 11/138 (8.0) | Ref |  |  |
| 1 day nonengagement | 22/211 (10.4) | 1.3 (0.7-2.6) | 0.446 |  |
| 2 days nonengagement | 13/68 (19.1) | 2.4 (1.1-5.1) | 0.022* |  |
| 3 days nonengagement | 58/191(30.4) | 3.8 (2.1-7.0) | <0.001* |  |
| **99DOTS patient- and provider-reported doses (N=608)**^e^ |  |  |  | 0.60 |
| 0 days nonengagement | 47/314 (15.0) | Ref |  |  |
| 1 day nonengagement | 28/212 (13.21) | 0.9 (0.6-1.4) | 0.572 |  |
| 2 days nonengagement | 5/14 (35.7) | 2.4 (1.1-5.1) | 0.023* |  |
| 3 days nonengagement | 24/68 (35.3) | 2.4 (1.6-3.6) | <0.001* |  |
| **Pill estimate (N=650)** |  |  |  | 0.57 |
| Taken as expected | 89/570 (15.6) | Ref |  |  |
| Excess of pills | 23/67 (34.3) | 2.2 (1.5-3.2) | <0.001* |  |
| Shortage of pills | 4/13 (30.8) | 2.0 (0.9-4.6) | 0.112 |  |
| **Four-day recall (N=650)** |  |  |  | 0.57 |
| 0 doses missed | 96/614 (15.6) | Ref |  |  |
| 1 dose missed | 10/19 (52.6) | 3.4 (2.1-5.4) | <0.001* |  |
| 2 doses missed | 4/9 (44.4) | 2.8 (1.3-6.0) | 0.007* |  |
| 3 or 4 doses missed | 6/8 (75.0) | 4.8 (3.1-7.4) | <0.001* |  |
| **Last missed dose question (N=650)** |  |  |  | 0.65 |
| Never skip medications | 61/483 (12.6) | Ref |  |  |
| Within the past week | 22/47 (46.8) | 3.7 (2.5-5.4) | <0.001* |  |
| 1-2 weeks ago | 7/34 (20.6) | 1.6 (0.8-3.3) | 0.172 |  |
| 2-4 weeks ago | 12/40 (30.0) | 2.4 (1.4-4.0) | 0.001* |  |
| 1-3 months ago | 13/35 (37.1) | 2.9 (1.8-4.8) | <0.001* |  |
| >3 months | 1/11 (9.1) | 0.7 (0.1-4.7) | 0.732 |  |

Ref=reference group

^a^Suboptimal adherence was defined as a yellow or green urine test result, as compared with a purple/blue result, which comprised adherence.

^b^Number of suboptimally adherent patients by urine testing divided by the number of patients in each alternate measure category.

^c^Prevalence ratio is a ratio of proportions—i.e., the proportion of patients with suboptimal adherence by urine testing in each category over the proportion of patients with suboptimal adherence in the reference category.

^d^AUCs are in relation to the categorical breakdown of variables presented in the table.

^e^We excluded 42 patients who were eligible for 99DOTS and recruited into the study, but whose 99DOTS enrollment date in the electronic system appeared to be after the home visit date.

*Indicates statistical significance at the 5% level

**Table S3. Operating characteristics of alternate tuberculosis medication adherence measures^a^ as compared to suboptimal adherence by urine isoniazid testing**

| Sample | Number of patients in sample (N) | Sensitivity^a^  % (95%CI) | Specificity^b^  % (95%CI) | Positive predictive value^c^  % (95%CI) | Negative predictive value^d^  % (95%CI) | Area under the receiver operating characteristic curve (AUC) |
| --- | --- | --- | --- | --- | --- | --- |
| 99DOTS patient-reported doses alone | 608 | 63 (58-67) | 68 (58-77) | 91 (87-93) | 27 (22-33) | 0.65 |
| 99DOTS patient- and provider-reported doses | 608 | 89 (86-92) | 28 (20-38) | 86 (82-89) | 35 (25-47) | 0.59 |
| Pill estimate | 650 | 90 (87-92) | 23 (16-32) | 84 (81-87) | 34 (24-45) | 0.57 |
| Four-day recall | 650 | 97 (95-98) | 17 (11-25) | 84 (81-87) | 56 (38-72) | 0.57 |
| Last missed dose question | 650 | 79 (75-82) | 47 (38-57) | 87 (84-90) | 33 (26-41) | 0.63 |
| Last missed dose question and four-day recall | 650 | 78 (75-82) | 49 (40-59) | 88 (84-90) | 33 (26-40) | 0.64 |
| Last missed dose question and pill estimate | 650 | 74 (70-78) | 53 (44-63) | 88 (85-91) | 31 (25-38) | 0.64 |
| Last missed dose question and four-day recall and pill estimate | 650 | 74 (70-77) | 55 (46-64) | 88 (85-91) | 31 (25-38) | 0.64 |

^a^For this analysis, alternate adherence measures were classified as binary variables, as described in Table 1.

**Table S4. Operating characteristics of alternate tuberculosis medication adherence measures as compared to nonadherence by urine testing – dose date and time correspondence (DDTC) analysis**

| Sample | Number of patients in sample (N) | Sensitivity  % (95%CI) | Specificity  % (95%CI) | Positive predictive value  % (95%CI) | Negative predictive value  % (95%CI) | Area under the receiver operating characteristic (ROC) curve |
| --- | --- | --- | --- | --- | --- | --- |
| DDTC^a^ analyses |  |  |  |  |  |  |
| Four-day recall | 641 | 100 (99—100) | 7 (2—16) | 90 (87—92) | 71 (29—96) | 0.53 |
| 99DOTS patient-reported doses alone | 597 | 70 (66—74) | 61 (48—72) | 93 (91—95) | 21 (18—25) | 0.65 |
| 99DOTS patient- and provider-reported doses | 597 | 85 (81—88) | 39 (28—52) | 91 (90—93) | 26 (20—33) | 0.62 |

^a^DDTC=dose date and time correspondence
