# Supplementary material for: Measuring Tuberculosis Medication Adherence: A Comparison of Multiple Approaches in Relation to Urine Isoniazid Metabolite Testing Within a Cohort Study in India
Source: Open Forum Infect Dis. 2021 Oct 17;8(11):ofab532. doi: 10.1093/ofid/ofab532 (PMC9088502; doi:10.1093/ofid/ofab532)
Supplement: ofab532_suppl_Supplementary_Materials_S1 [file ofab532_suppl_Supplementary_Materials_S1.docx]

**Supplementary Text**

**Supplement to:**

*Measuring tuberculosis medication adherence: a comparison of multiple approaches in relation to urine isoniazid metabolite testing within a cohort study in India*

**Authors:** Ramnath Subbaraman, Beena E. Thomas, J. Vignesh Kumar, Maya Lubeck-Schricker, Amit Khandewale, William Thies, Misha Eliasziw, Kenneth H. Mayer, Jessica E. Haberer

**Correspondence:** Beena E. Thomas, Department of Social and Behavioural Research, ICMR- National Institute for Research in Tuberculosis, No. 1, Mayor Sathiyamoorthy Road, Chetpet, Chennai – 600 031, India ([beenaelli09@gmail.com](mailto:beenaelli09@gmail.com)). Phone: +91-44-2836-9525

**Methods**

*Further details on the operating characteristics of urine isoniazid testing*

Although several researchers have used the IsoScreen test or the Arkansas method (on which the IsoScreen test is based) in clinical studies [1,2] or small validation studies [3–7], we identified only two studies that specifically evaluated the proportion of participants who have positive test results at different time intervals after verified ingestion of a last isoniazid medication dose. For interested readers, we describe background data from these two studies that directly informed our understanding of the operating characteristics of the IsoScreen test [8,9]. Both studies evaluated color changes in urine test results 24 hours, 48 hours, and 72 hours after study participants last took a dose of isoniazid. These studies therefore parallel changes that would be expected in IsoScreen test results when patients experience non-adherence—that is, when they miss 1, 2, or 3 or more daily TB medication doses. Notably, one of these studies evaluated changes in IsoScreen test results over time among healthy participants who took a single dose of isoniazid [8], while another evaluated these changes among participants taking treatment for active or latent TB after directly observing the last dose of their treatment course [9]. To estimate the sensitivity (for adherence) and specificity (for nonadherence) of urine testing, we combined findings from these studies by estimating the average (mean) proportion of participants with each type of color change for each time interval, as shown in Table S1.

*Operating characteristics for suboptimal adherence by urine testing*

We defined “suboptimal adherence” as comprising a yellow or green urine test result (compared to a purple/blue result), which suggests missed doses for 48 hours or more. For our definition of suboptimal adherence by urine testing, the test’s sensitivity for doses taken within 24 hours is 85% and its specificity for not having taken dose for 72 hours is 100% (Table S1). As such, our definition of suboptimal adherence has a <15% chance of misclassifying participants who took a dose within 24 hours as being suboptimally adherent. Although our definition of nonadherence has risk of misclassifying a small percentage of participants with true nonadherence, the risk of misclassifying true adherence by our definition of suboptimal adherence is more concerning given that most participants were probably taking their medications at the time of the home visit. As a result, our definition of suboptimal adherence has the risk of misclassifying a larger number of participant results. For this reason, we prioritized findings of analyses using nonadherence by urine testing as an outcome in the main manuscript, whereas we present findings of analyses for the outcome of suboptimal adherence by urine testing below in the supplementary text and tables.

*Dose date and time correspondence (DDTC) analysis*

Our methods for the DDTC analysis for 99DOTS were described in detail in a prior manuscript [10]. The goal of the DDTC analysis was different from the predictive analysis, in that the DDTC analysis aimed to more precisely evaluate whether the exact reported adherence history—based on the dates and times participants reported taking doses in the three days prior to the home visit—accurately reflected medication ingestion in comparison to the urine test result. As such, the DDTC approach takes into account the operating characteristics of the urine test, and it is only possible to do this analysis for the two alternate adherence measures (99DOTS and four-day recall) that captured the times that participants reported taking doses in the three days prior to the home visit.

For the DDTC analysis, we identified time windows in which the urine isoniazid test results could be variable if doses were only taken within those time windows. We call these time windows “gray zone” intervals. For example, we assumed that it takes a few hours for isoniazid to be absorbed by the gastrointestinal tract after ingestion of medication, such that if a participant only took a dose within 6 hours before the home visit (without taking any other doses in the preceding 48 hours), the urine test might have a variable result (i.e., yellow, green, or purple/blue color change). Similarly, based on the test’s operating characteristics reported in Table S1, urine test results are also variable 48—72 hours after last ingestion of isoniazid.

As such, we excluded from analysis test results for participants whose reported doses, as per 99DOTS or four-day recall, were only taken within gray zone timings. In other words, participant data were excluded if doses were reported as having been taken <6 hours and 48—72 hours prior to the home visit, *and* no doses were reported as having been taken 6–48 hours before the home visit. For the 99DOTS DDTC analysis, we excluded data for 42 participants whose 99DOTS enrollment date in Nikshay (the national TB program’s electronic medical record) appeared to be after the home visit date, and we excluded data for an additional 11 participants whose reported doses taken fell within gray zone timings (in total, about 8% of participant test results). For the four-day recall DDTC analysis, we excluded data for 9 participants whose reported doses fell within gray zone timings (about 1% of participant test results).

To be clear, the gray zone timings do not reflect limitations in the operating characteristics of the urine test itself. Gray zone timings only reflect that the urine test result could be variable (and therefore not an optimal comparator) for very specific dose-taking case scenarios in the DDTC analysis. Since the goal of the DDTC analysis is to determine if an alternative adherence measure is telling the exact “truth” about dose-taking in the prior three days, we used *each participant’s reported adherence pattern (not their urine test result)* to exclude participants from the analysis.

For the DDTC analysis, we only used the definition of nonadherence by urine testing, in which “adherence” comprised a purple/blue or green result (indicating a dose taken in the last 6—48 hours) and “nonadherence” comprised yellow result (indicating no doses taken for >72 hours). For 99DOTS and four-day recall, participants who reported taking at least one dose 6—48 hours before the home visit were classified as “adherent” by each measure. Participants who reported that the last dose was taken >72 hours before the visit were classified as “nonadherent” by each measure. 99DOTS also allows healthcare providers to report doses after calling patients to evaluate whether they took a dose on a given day. As such, in our prior study we analyzed 99DOTS’ operating characteristics using only patient-reported doses alone and using both patient- and healthcare provider-reported doses [10]. We report 99DOTS operating characteristics for both patient-reported doses alone and patient- and healthcare provider-reported doses from our prior study again here, to allow comparison to the DDTC analysis findings for four-day recall.

**Results**

*Prevalence ratios of alternate adherence measures in relation to the outcome of suboptimal adherence by urine testing*

All adherence measures had some categories with statistically significant increases in prevalence ratios of suboptimal adherence by urine testing (Table S2). For 99DOTS, categories in which participants had 2 or 3 days of nonengagement had statistically significantly increased prevalence ratios of suboptimal adherence by urine testing. For the pill estimate, observed pill excess had a statistically significantly increased prevalence ratio for suboptimal adherence by urine testing. For four-day recall, the magnitude of the association with suboptimal adherence by urine testing was generally greater with higher reported missed doses. For the last missed dose question, in general, the magnitude of the association with suboptimal adherence by urine testing was higher the more recently participants reported missing doses.

*Accuracy and operating characteristics of the alternate adherence measures in relation to the outcome of suboptimal adherence by urine testing*

For suboptimal adherence by urine testing, 99DOTS using patient-reported doses alone and the last missed dose question had the highest AUCs, regardless of whether these alternate adherence measures were considered as ordinal or nominal variables (Table S2) or as binary variables (Table S3). With regard to operating characteristics, for suboptimal adherence by urine testing, 99DOTS using patient-reported doses alone had the highest specificity followed by the last missed dose question, whereas 99DOTS using patient- and provider-reported doses, the pill estimate, and four-day recall had considerably lower specificity (Table S3). In contrast, for suboptimal adherence by urine testing, four-day recall, the pill estimate, and 99DOTS using patient- and provider-reported doses had the highest sensitivity, whereas the last missed dose question had lower sensitivity and 99DOTS using patient-reported doses alone had considerably lower sensitivity. For suboptimal adherence by urine testing, adding four-day recall and the pill estimate to the last missed dose question mildly increased specificity for identifying suboptimally adherent participants while also mildly decreasing sensitivity.

*Dose date and time correspondence analysis findings*

In the DDTC analysis, four-day recall had considerably lower specificity and lower AUC compared to analyses with four-day recall using the predictive approach (Table S4). The 99DOTS DDTC analyses are presented below for comparison; these findings were presented and discussed in detail in a previous manuscript [10].

**References**

1. Whitfield R, Cope GF. Point-of-care test to monitor adherence to anti-tuberculous treatment. Ann Clin Biochem **2004**; 41:411–413.

2. Nackers F, Huerga H, Espié E, et al. Adherence to self-administered tuberculosis treatment in a high HIV-prevalence setting: a cross-sectional survey in Homa Bay, Kenya. PLoS One **2012**; 7:e32140.

3. Guerra RL, Conde MB, Efron A, et al. Point-of-care Arkansas method for measuring adherence to treatment with isoniazid. Respir Med **2010**; 104:754–757.

4. Schraufnagel DE, Stoner R, Whiting E, Snukst-Torbeck G, Werhane MJ. Testing for isoniazid. An evaluation of the Arkansas method. Chest **1990**; 98:314–316.

5. Schmitz KE, Hovell MF, Wong CA, et al. The reliability and practicality of the Arkansas method assay of isoniazid adherence. Clin Nurs Res **2010**; 19:131–143.

6. Perry S, Hovell MF, Blumberg E, et al. Urine testing to monitor adherence to TB preventive therapy. J Clin Epidemiol **2002**; 55:235–238.

7. Nicolau I, Tian L, Menzies D, Ostiguy G, Pai M. Point-of-care urine tests for smoking status and isoniazid treatment monitoring in adult patients. PLoS One **2012**; 7:e45913.

8. Elizaga J, Friedland JS. Monitoring compliance with antituberculous treatment by detection of isoniazid in urine. Lancet **1997**; 350:1225–1226.

9. Soobratty MR, Whitfield R, Subramaniam K, et al. Point-of-care urine test for assessing adherence to isoniazid treatment for tuberculosis. Eur Respir J **2014**; 43:1519–1522.

10. Thomas BE, Kumar JV, Chiranjeevi M, et al. Evaluation of the accuracy of 99DOTS, a novel cellphone-based strategy for monitoring adherence to tuberculosis medications: Comparison of digital adherence data with urine isoniazid testing. Clin Infect Dis **2020**; 71:e513–e516.
